# Recent advances in the search for a targeted immunomodulatory therapy for primary Sjögren’s syndrome

**DOI:** 10.12688/f1000research.19842.1

**Published:** 2019-08-29

**Authors:** David L. Leverenz, E. William St. Clair

**Affiliations:** 1 Department of Medicine, Division of Rheumatology and Immunology, School of Medicine, Duke University, 40 Duke Medicine Circle, Durham, NC, 27110, USA

**Keywords:** Sjögren's Syndrome, Sicca, Rheumatologic diseases

## Abstract

Primary Sjögren’s syndrome is a chronic autoimmune disease characterized by salivary and lacrimal gland dysfunction, leading to substantial morbidity and reduced quality of life. Many patients with primary Sjögren’s syndrome also have extraglandular systemic complications, some of which can be organ- or life-threatening. Over the last decade, numerous targeted immunomodulatory therapies for primary Sjögren’s syndrome have failed to show a benefit in clinical trials, and as yet no disease-modifying therapy has been approved for this disease. Herein, we provide an updated review of the clinical trial landscape for primary Sjögren’s syndrome and the numerous efforts to move the field forward, including the development of new classification criteria and outcome measures, the results of recent clinical trials in this field, the challenges faced in the search for effective therapies, and the expanding pipeline of novel therapies under development.

## Introduction

Primary Sjögren’s syndrome (pSS) is a chronic autoimmune disease characterized by salivary and lacrimal gland dysfunction, leading to substantial morbidity and reduced quality of life. Patients with pSS not only experience the consequences of exocrine gland deficiency but also may manifest systemic complications, including profound fatigue, rash, arthritis, interstitial lung disease, nephritis, and neuropathy. They also carry an increased risk for non-Hodgkin’s B-cell lymphoma. Since the description of this syndrome by Henrik Sjögren in 1933 ^1^, extensive efforts have been made to classify pSS more precisely, understand its pathogenesis, and develop effective treatments. Despite these efforts, the management of pSS has not advanced much beyond alleviating symptoms of glandular dysfunction and controlling any systemic manifestations by using immunomodulatory agents borrowed from the therapeutic armamentarium of other autoimmune diseases, such as rheumatoid arthritis (RA) and systemic lupus erythematosus (SLE). To date, there is no proven disease-modifying therapy for pSS.

There are several reasons why disease-modifying therapies for Sjögren’s syndrome have struggled to reach the clinic. First, the field has been slow to embrace the development of novel targeted therapies for pSS compared with the extensive work devoted to finding novel therapies for RA and other types of inflammatory arthritis. Until recently, a prevailing view among many pharmaceutical and biotechnology companies was the misperception that pSS was a relatively benign condition and not an area ripe for investment. The outlook for developing new drugs for the treatment of pSS has changed in the last several years with the recognition that glandular dysfunction is more than a nuisance and that extraglandular disease may be fraught with serious complications. Second, the development of disease-modifying therapies for pSS has been more challenging than was expected when compared with the relative success in finding new therapies for RA and other systemic inflammatory diseases. The results of two randomized, placebo-controlled trials showing that rituximab lacks clinical efficacy in pSS have been sobering for the field.

The failure of rituximab and many other immunomodulatory therapies for pSS has illuminated a number of challenges in the design of clinical trials for pSS, in particular regarding patient selection, assessment of treatment efficacy, and the identification of viable therapeutic targets ( Table 1). What is the appropriate strategy for selecting the study population in clinical trials? To this end, the American College of Rheumatology (ACR) and the European League Against Rheumatism (EULAR) recently revised the classification criteria for Sjögren’s syndrome. However, this scheme, like previous criteria, tends to capture a clinically heterogeneous group of patients in varying stages of disease. It is also uncertain how to identify patients with reversible glandular dysfunction, which is important for testing a new drug with potential efficacy for improving tear and salivary flow. To increase the likelihood of detecting an improvement in glandular function, many clinical trials of late have required eligible subjects to show residual stimulated whole salivary flow.

**Table 1.** Current challenges in clinical trials of new therapies for primary Sjögren’s syndrome.

**Selection of study population** • Identifying patients with reversible glandular dysfunction • Individuals with diverse extraglandular features may not respond alike to a particular therapy. • Lack of patient stratification (for example, defined on the basis of mechanism or predictive biomarker) may obscure detection of therapeutic response. **Assessment of treatment efficacy** • Limited sensitivity to change of patient-reported symptom scales • Drawbacks of collapsing a multi-domain index, such as the EULAR Sjögren’s syndrome Disease Activity Index (ESSDAI), into a single measure of disease activity for use as a primary endpoint • Lack of reliable biomarkers of disease activity to substantiate treatment efficacy **Identification of therapeutic targets** • Disease-modifying agents (including biologics) effective in other autoimmune diseases have not been effective in primary Sjögren’s syndrome. • Monotherapy may be inadequate to control disease activity. (For example, combinations of drugs targeting different aspects of the pathologic response may be required.) • More work is needed on targeting the interaction of immune cells with acinar and ductal epithelial cells to potentially reverse glandular dysfunction.

The identification of effective therapies for pSS will depend on the validity, reliability, responsiveness, ceiling effects, and floor effects of the outcome measures used in the clinical trials. Recent and ongoing trials have relied mostly on the EULAR Sjögren’s Syndrome Disease Activity Index (ESSDAI) and EULAR Sjögren’s Syndrome Patient-Reported Index (ESSPRI), which are validated outcome measures for the assessment of systemic activity and patient symptoms, respectively. The ESSDAI is a multi-domain index that collapses a weighted score from each of 10 different clinical items, a hematologic item, and a biological item into a single score. The ESSDAI is considered the gold standard for assessing systemic disease activity in clinical trials, and a minimum score of 5 is usually set as an eligibility requirement in clinical trials testing therapies aimed at reducing systemic disease. This approach may be problematic in a clinically heterogeneous disease such as pSS. One potential pitfall is that patients with a diverse array of organ system involvement may not have the same chance of improving their score owing to differences in the sensitivity of that domain or item’s scale to change and the inherent responsiveness of a given domain or item to therapy. In addition, changes between the pre-defined categories of absent, low, moderate, or high disease activity may underestimate or overestimate the extent of improvement, depending on the baseline assessment.

Another reason for the slow progress in bringing new drugs into the clinic may relate to the strategy for choosing a therapeutic target. Therapies tested thus far in pSS have been based mostly on a track record of success in SLE (for example, belimumab and rituximab) or RA (for example, rituximab and abatacept) owing to the overlap in clinical and pathologic features among these autoimmune diseases. This strategy has obvious drawbacks, as our recent experience has taught us. pSS likely has unique immune mechanisms at play, particularly in light of the important role of epithelial cells in the pathogenesis of this disease. New therapies should be considered on the basis of a strong scientific rationale relevant to the pathogenesis of pSS.

## Clinical trials in primary Sjögren’s syndrome: coming up dry

Hydroxychloroquine, which has been proven effective for the treatment of SLE and RA, has been prescribed for patients with pSS with the goal of improving symptoms of fatigue and arthritis. However, such a benefit was not confirmed in a randomized clinical trial of hydroxychloroquine therapy for pSS (Randomized Evaluation of hydroxychloroquine in primary **Sjögren’s** syndrome, known as the JOQUER trial) ^2^. Prior to that study, a number of open-label and retrospective studies had demonstrated possible benefits of hydroxychloroquine for reducing fatigue, arthralgia, myalgia, and dryness in pSS ^3– 6^. However, the JOQUER trial failed to show a significant impact of hydroxychloroquine therapy on any of these patient-reported measures. As discussed later, in pSS, indices of patient-reported symptoms are not sensitive to change over time and therefore the outcome measures used in the JOQUER trial may have underestimated the true effect of hydroxychloroquine treatment on improving fatigue and arthralgia.

Recently, rituximab therapy failed to show a benefit in two clinical trials despite the compelling evidence supporting the key roles of B-cells in disease pathogenesis, such as the high prevalence of autoantibodies and hypergammaglobulinemia, the presence of germinal center–like structures in salivary gland biopsies, the increased risk of non-Hodgkin’s B-cell lymphoma, and genetic studies linking the risk for pSS with polymorphisms in genes critical for B-cell development ^7, 8^. The optimism for targeting B-cells was fueled by promising results in early pilot studies of rituximab in pSS ^9– 11^. However, these results were not confirmed in the two large trials. The Tolerance and Efficacy of Rituximab in pSS (TEARS) trial evaluated the effect of rituximab therapy on the symptoms of pSS, employing as outcome measures four patient-reported visual analogue scales (VASs): global disease, pain, fatigue, and dryness ^12^. The proportions of patients with a treatment response, as defined by an improvement of 30 mm or more in two out of four VAS outcome measures, were not significantly different between the rituximab and placebo groups. There were some suggestions of clinical efficacy, as some pre-specified secondary analyses showed a significant difference between the rituximab and placebo groups at weeks 6 and 16, particularly in the fatigue domain.

The Trial of Anti-B cell Therapy in Patients with pSS (TRACTISS) began enrollment later and called for two courses of rituximab (or placebo) and used a primary endpoint defined as the proportion of patients achieving a 30% reduction in fatigue or oral dryness by VAS at 48 weeks ^13^. Secondary outcomes included measures of salivary and lacrimal flow. The results of this trial also showed no significant differences between rituximab and placebo for the primary endpoint or any of the secondary endpoints, except for a small improvement in unstimulated salivary flow rate in the group that received rituximab.

Lymphotoxin beta (LT-β) is required for the formation of lymph nodes and germinal centers and thus is an alternative approach for targeting B cell–mediated immune responses. LT-α induces the secretion of interferon and chemokines, and LT-α/LT-β heterodimers stimulate the development of ectopic germinal center–like structures ^14^. Animal models had previously shown that blocking LT-β prevents lymphoid organization in salivary glands and improves their function ^15^, and human studies had shown that LT-β is upregulated in salivary gland tissue of patients with pSS ^16^. However, in a randomized, placebo-controlled, phase II clinical trial, baminercept, an LT-β receptor fusion protein, failed to positively impact any of the clinical measures of disease activity despite evidence of a biological effect ^17^.

Abatacept, another biologic investigated in pSS, mimics the activity of CTLA-4 and prevents T-cell co-stimulation. It is approved for the treatment of RA and psoriatic arthritis in adults and juvenile idiopathic arthritis in children and had shown promise in open-label studies of patients with pSS ^18, 19^. The results of a randomized, placebo-controlled phase III trial of abatacept in 187 patients with pSS were reported at a scientific meeting and indicated that 24 weeks of abatacept therapy was no better than placebo for improving any of the clinical outcome measures, including the ESSDAI or ESSPRI ^20^. Ongoing trials in the Sjögren’s syndrome area investigating the agents mentioned above as well as others are listed in Table 2.

**Table 2.** Ongoing clinical trials in the ClinicalTrials.gov database evaluating targeted therapies for primary Sjögren’s syndrome.

ClinicalTrials. gov identifier	Molecule	Phase	Targeted number of subjects	Selected eligibility criteria	Primary endpoint	Status
NCT02631538	Belimumab (anti-BAFF) and Rituximab (anti-CD20)	II	79	ESSDAI ≥ 5 Baseline unstimulated salivary flow > 0 mL/min or stimulated salivary flow > 0.05 mL/min	SAEs and AESIs	Active, not recruiting
NCT02962895	VAY736 (anti-BAFF-R)	II	180	ESSDAI ≥ 6 from seven selected domains	Unspecified disease assessment	Recruiting
NCT03627065	Paraclisib (PI3Kδ inhibitor)	II	12	ESSDAI ≥ 5 SGUS ≥ 2	SGUS	Recruiting
NCT02067910	Abatacept (CTLA4 Ig)	III	80	ESSDAI ≥ 5 + parotid gland biopsy	ESSDAI	Active, not recruiting
NCT02915159	Abatacept (CTLA4 Ig)	III	253	ESSDAI ≥ 5	ESSDAI	Active, not recruiting ^a^, ^20^
NCT03905525	CFZ533 (anti-CD40)	II	260	Stimulated whole salivary flow ≥ 1 mL/min AND ESSDAI ≥ 5 from eight selected domains OR ESSPRI fatigue or dryness sub-scores ≥ 5	ESSDAI and ESSPRI	Not yet recruiting
NCT03100942	Filgotinib (JAK1) Lanraplenib (Syk) Tirabrutinib (BTK)	II	152	ESSDAI ≥ 5	Protocol-specified response criteria	Active, not recruiting
NCT01988506	Low-dose IL-2 (to induce Treg cells)	II	132 ^b^	Not reported	% Treg cells in blood	Recruiting

Accurate as of June 20, 2019. Excluded trials are listed as unknown, completed, withdrawn, or terminated. Also excluded are trials without an update in the ClinicalTrials.gov database in the last two years. ^a^Preliminary results from this study were reported in abstract form at the European League Against Rheumatism (EULAR) national meeting in 2019 and discussed in the text of this article ^20^. ^b^Trial also includes 13 other autoimmune diseases. AESI, adverse event of special interest; BAFF, B-cell activating factor; BAFF-R, B-cell activating factor receptor; BTK, Bruton’s tyrosine kinase; CTLA-4, cytotoxic T-lymphocyte associated protein-4; ESSDAI, EULAR Sjögren’s Syndrome Patient Disease Activity Index; ESSPRI, EULAR Sjögren’s Syndrome Patient-Reported Index; IL-2, interleukin 2; JAK, Janus kinase; PI3Kδ, phosphatidylinositol-3 kinase delta; SAE, serious adverse event; SGUS, salivary gland ultrasound score; Treg, regulatory T.

## Selection of patients for clinical trials

In a clinical trial, the likelihood of detecting a treatment response depends in part on the characteristics of the study population. First, it is important to enroll patients with the disease of interest, avoiding the selection of patients with a similar set of symptoms resulting from a different disease process. The identification of eligible patients ideally should be relatively straightforward without reliance on specialized testing. The latest set of classification criteria for Sjögren’s syndrome are endorsed by both the ACR and EULAR ^21, 22^. The 2002 American-European Consensus Group (AECG) ^23^ and the 2012 Sjögren’s International Collaborative Clinical Alliance Cohort (SICCAC) ^24^ criteria preceded this most recent scheme. Both the 2002 AECG and 2012 SICCAC criteria depended on objective measures of dryness, autoantibody testing, and histopathology for a patient to be classified as having pSS. Although these two classification criteria had sensitivities and specificities greater than 90% for the classification of pSS, they each had issues ^25, 26^. For example, the 2002 AECG criteria relied on tests of salivary gland function unavailable in most clinics, and the 2012 SICCAC criteria were felt to be too invasive because of the requirement for corneal staining or a lip biopsy.

These concerns led to the development of the 2016 ACR/EULAR criteria, which are meant to be applied to patients with signs or symptoms suggestive of Sjögren’s syndrome. Table 3 compares the specific elements of these various classification schemes. Similar to the 2002 AECG and 2012 SICCAC criteria, the 2016 ACR/EULAR criteria rely primarily on objective tests for the classification of Sjögren’s syndrome. However, the new system differs from its predecessors by using a weighted score of each element, updating the ocular staining score domain to a more specific threshold, and eliminating anti-La/SS-B antibodies from the criteria.

**Table 3.** Comparison of the 2002 AECG, 2012 SICCAC, and 2016 ACR/EULAR classification criteria for primary Sjögren’s syndrome.

	2002 AECG	2012 SICCAC	2016 ACR/EULAR
Criteria	1. Pathology showing focal lymphocytic sialadenitis with a focus score ≥ 1 2. Positive test for anti-Ro/SS-A or anti- La/SS-B antibodies 3. Ocular signs (Schirmer I test ≤ 5 mm per 5 min or Rose Bengal score ≥ 4 by the van Bijsterveld scoring system) 4. Objective evidence of salivary gland involvement by salivary scintigraphy, parotid sialography, or unstimulated salivary flow rate ≤ 0.1 mL/min 5. Ocular symptoms 6. Oral symptoms	1. Pathology showing focal lymphocytic sialadenitis with a focus score ≥ 1 2. Positive anti-Ro/SS-A OR anti- La/SS-B antibodies OR a positive test for rheumatoid factor and antinuclear antibodies ≥ 1:320 3. Ocular staining score ≥ 3	1. Pathology showing focal lymphocytic sialadenitis with a focus score ≥ 1 (worth 3 points) 2. Positive anti-Ro/SS-A antibodies (worth 3 points) 3. SICCA ocular staining score ≥ 5 or Rose Bengal score ≥ 4 by the van Bijsterveld scoring system (worth 1 point) 4. Schirmer I test ≤ 5 mm per 5 min (worth 1 point) 5. Unstimulated whole salivary flow less than 0 ≤ 0.1 mL/min (worth 1 point)
Rules for classification as pSS	Patients must have at least four of the above six criteria provided that: 1. Item 1 (pathology) is positive OR 2. Item 2 (serology) is positive OR 3. Any three of the four objective items (1–4) are positive	Patients must have at least two of the above three criteria.	Patients must have a total score ≥ 4 when the weights from the five criteria items above are summed AND 1. At least one symptom of ocular/oral dryness OR 2. An extraglandular manifestation of pSS as defined by the ESSDAI

ACR, American College of Rheumatology; AECG, American-European Consensus Group; ESSDAI, EULAR Sjögren’s syndrome Disease Activity Index; EULAR, European League Against Rheumatism; pSS, primary Sjögren’s syndrome; SICCA, Sjögren’s International Collaborative Clinical Alliance; SICCAC, Sjögren’s International Collaborative Clinical Alliance Cohort.

The removal of anti-La/SS-B antibodies from the criteria is based on recent evidence that anti-La/SS-B antibodies alone are not useful for classification purposes. In a study published in 2015, Baer *et al*. analyzed the autoantibody profile of over 3,000 participants in the Sjögren’s International Collaborative Clinical Alliance (SICCA) registry and found that only 2% of patients were positive for anti-La/SS-B without the presence of concomitant anti-Ro/SS-A antibodies and that no difference in the clinical phenotype was evident between these patients who were positive for anti-La/SS-B antibody alone and patients testing negative for both anti-Ro/SS-A and anti-La/SS-B antibodies ^27^. In other words, the presence of anti-La antibodies alone lacked specificity for the diagnosis of pSS.

There is a high concordance between the previously published classification criteria and the 2016 ACR/EULAR criteria ^28– 30^. However, an important change from older criteria is that the latest criteria give credit for the presence of an extraglandular manifestation. For example, a patient with reduced tear flow by Schirmer’s testing, normal salivary flow, a small fiber neuropathy, and positive anti-Ro/SS-A antibodies would not have met previously published criteria. According to the 2016 ACR/EULAR criteria, however, the strong weighting of a positive test for anti-Ro/SS-A antibody and the presence of an extraglandular manifestation meets the threshold for classification as pSS. Similarly, a patient with reduced tear flow by Schirmer’s testing and a small fiber neuropathy would meet these new classification criteria if they had a positive labial salivary gland biopsy, even in the absence of anti-Ro/SS-A antibodies. This fine-tuning of the classification criteria allows patients with a predominant extraglandular feature of pSS to be included in clinical trials.

Whether these criteria may efficiently identify patients with early disease remains a question. Antibodies to salivary protein 1 (SP1), carbonic anhydrase 6 (CA6), and parotid secretory protein (PSP), which have been touted as early biomarkers of pSS, are reported to be present in about half the patients with this disease, including many patients negative for anti-Ro/SSA and anti-La/SS-B ^31, 32^. However, the clinical significance of these antibodies remains uncertain, as demonstrated in a study by Karakus *et al*., who compared the autoantibody profile among three groups: 46 patients with pSS, 14 patients with dry eyes but without pSS (non-SS dry eye), and 25 controls ^33^. While 61% of the patients with pSS had at least one positive test for anti-SP1, CA6, or PSP antibodies, so did 50% of the non-SS dry eye patients and 16% of controls. At this stage, the definition of “early Sjögren’s syndrome” remains elusive and its elucidation will require large cohort studies with adequately long follow-up.

Salivary gland ultrasound has emerged as an intriguing diagnostic tool in the evaluation of patients with pSS. It has, an estimated sensitivity and specificity ranging from 45.8% to 91.6% and from 73% to 98.1%, respectively ^34^. In 2016, Le Goff *et al*. reported that ultrasound imaging of the parotid glands might improve the sensitivity of the 2016 ACR/EULAR classification criteria ^29^. This study assessed 290 patients with possible pSS and found that 125 (43%) met ACR/EULAR criteria. However, an additional seven patients strongly suspected to have pSS by clinical assessment were found to have abnormalities by salivary gland ultrasonography. Each of these patients had either a positive anti-Ro/SS-A antibody or an abnormal salivary gland biopsy but because of a normal Schirmer’s test, normal van Bijsterveld score for ocular dryness, or normal unstimulated whole salivary flow rate did not meet the 2016 ACR/EULAR criteria. Overall, the authors found that adding salivary gland ultrasound to the ACR/EULAR criteria increased sensitivity from 87.4% to 91.1% and only slightly decreased specificity, from 95.4% to 93.8%. There may be a practical limitation of including salivary gland ultrasonography in future classification schemes unless this technology becomes more widespread.

An extensive body of literature developed over several decades has provided a standardized approach for identifying and quantifying focal lymphocytic sialadenitis, the defining histologic feature of pSS ^35– 38^. This histopathologic evaluation results in a focus score, in which a focus is defined as a cluster of 50 or more lymphocytes per 4 mm ^2^ of tissue, and a focus score of greater than or equal to 1 meets the histopathologic threshold for supporting the classification of pSS ( Table 3). In 2015, Costa *et al*. reported that the histopathologic interpretations of labial salivary gland biopsies by expert and local pathologists in the TEARS study showed poor inter-observer reliability ^39^. In particular, local pathologists did not routinely follow a standardized protocol for determining a focus score, such that the focus score was often overestimated. In response to these and other issues, the EULAR Sjögren’s syndrome Study Group recently established consensus guidelines to improve the standardization of the acquisition and interpretation of tissue histopathology for clinical trials ^40^. These guidelines clarify important points about tissue requirements, the calculation of a focus score, identification of germinal centers, and reporting standards. Importantly, these guidelines called for a change in the methodology used to count foci of sialadenitis adjacent to areas of atrophy, duct dilation, and fibrosis. Previously, these areas have not been counted as part of the focus score, because many biopsy specimens show non-specific patterns of inflammation with mixed lymphocytic and plasma cell infiltrates as a normal response to duct damage that occurs with aging and other non-autoimmune factors ^38^. According to the new EULAR recommendations, if focal lymphocytic sialadenitis consistent with pSS is found to be present elsewhere in the biopsy, these areas of inflammation near damaged ducts should now be counted as a part of the focus score in order to better quantify the degree of damage, reduce the risk of bias in the interpretation, and improve reproducibility ^40^. If the results of labial salivary gland biopsies are used for determining eligibility in clinical trials, they should be rendered by a central pathology reading core to minimize misinterpretation and inclusion of subjects without bona fide Sjögren’s syndrome.

## Outcome measures in clinical trials

A major focus of discussion about clinical trials in pSS has centered on the adequacy of outcome measures for detecting a treatment response. The primary outcome measures used in the JOQUER, TEARS, and TRACTISS trials relied on patient-reported scales of glandular dryness, pain, and fatigue. Many investigators have questioned whether the failure of these trials to find a treatment response in the active arm could be due, at least in part, to a lack of sensitivity of the outcome measures. Clinical trials in pSS have shifted almost exclusively to using the ESSDAI as the primary outcome measure because the main objective of these studies has been to determine whether the experimental treatment affects systemic disease activity ( Table 2). The ESSDAI, which was developed in 2010 by the EULAR Sjögren’s Syndrome Task Force, quantifies the extent of systemic disease activity in 12 different domains: constitutional, lymphadenopathy, glandular enlargement, articular, cutaneous, pulmonary, renal, muscular, peripheral nervous system, central nervous system, hematologic, and biological (hypocomplementemia, hypergammaglobulinemia, or cryoglobulinemia) ^41^. Each domain score in the ESSDAI is derived from physician-entered data and objective diagnostic testing. In a multi-center validation study of the ESSDAI, Seror *et al*. demonstrated that the ESSDAI correlated poorly with patient-reported measures (including the ESSPRI) and that the ESSDAI is significantly more sensitive to change over time ^42^. For this reason, the ESSDAI may be better at detecting changes in disease activity than patient-centered measures. However, the ESSDAI has some potential drawbacks, as mentioned earlier. Some domains (for example, arthritis and constitutional) are more commonly scored than others, and less commonly scored items such as the renal and muscular domains will be under-represented in the evaluation of outcomes. In addition, some domains are easier to accurately assess than others. For example, lung disease may be easier to follow using serial pulmonary function tests and chest computed tomography scans than tracking the progress of peripheral neurologic disease by examination and electrophysiologic testing. Furthermore, some patients may exhibit a clinically meaningful improvement in a single clinical domain of the ESSDAI without showing a change in the composite score if the score from another domain, such as the glandular item or hematologic item, worsens with relatively less clinical impact.

Many of the potential pitfalls of the ESSDAI and ESSPRI arise from the substantial clinical heterogeneity of pSS. Several lines of evidence suggest that this clinical heterogeneity may be driven by non-overlapping disease mechanisms. For example, in 2016, Nishikawa *et al*. identified CXCL13, TNF-R2, CD48, B-cell activating factor (BAFF), and PD-L2 as potential serum biomarkers in patients with pSS. Intriguingly, serum levels of CXCL13 correlated positively with the lymphadenopathy, glandular, and pulmonary domains of the ESSDAI, while serum levels of CXCL13, TNF-R2, and CD48 correlated positively with the biological domain of the ESSDAI; and serum levels of TNF-R2 correlated with low uptake on salivary gland scintigraphy ^43^. In 2018, Kanne *et al*. found that patients with pSS and extraglandular involvement had increased serum levels of high mobility group box chromosomal protein 1, a pro-inflammatory cytokine also associated with renal involvement in SLE ^44^. A study published in 2018 by James *et al*. correlated markers of B-cell activity with sub-components of the ESSDAI score in 533 patients in the UK PSS Registry and found that serum levels of BAFF correlated with the peripheral nervous system domain of the ESSDAI but that serum levels of β-2 microglobulin and free light chains correlated with the cutaneous, renal, and biological domains ^45^. More work is needed to determine whether serum biomarkers can be used to subset disease in a rational way or inform the assessment of treatment response.

Data from the TEARS and TRACTISS trials have been used to investigate the potential utility of ultrasound as an outcome measure in pSS. Salivary gland ultrasound can identify characteristic changes in parenchymal volume, echogenicity, homogeneity, and vascularity ^46, 47^. In the TEARS trial, 28 patients underwent testing by salivary gland ultrasound at baseline and then six months after receiving rituximab (14 patients) or placebo (14 patients). There was a statistically significant improvement in the rituximab-treated compared with the placebo-treated participants in ultrasound parameters of parotid parenchyma echostructure (50% versus 7%, *P* = 0.03) and a trend toward an improvement in submandibular gland echostructure (36% versus 7%, *P* = 0.16) ^48^. In a similar study, 52 patients in the TRACTISS trial underwent salivary gland ultrasound testing at baseline and at weeks 16 and 48 after treatment with rituximab (26 patients) or placebo (26 patients). That study also found a statistically significant improvement in salivary gland ultrasound echostructural features in the patients who received rituximab compared with placebo ^49^. Although these studies in pSS suggest that rituximab therapy has a biological impact on salivary gland abnormalities by ultrasound, the relevance of these findings to disease-modification remains to be determined.

## Identifying mechanistically based therapeutic targets

The pathogenesis of pSS, like that of many rheumatologic diseases, involves a complex interplay between many components in the innate and adaptive immune system ^50^. At present, several cellular components and signaling molecules and pathways—including B-cells, co-stimulatory pathways, PI3Kδ, and interferon—have emerged as potential targets for therapeutic intervention.

Despite the failure of rituximab therapy to show a benefit in the TEARS and TRACTISS trials, B-cells remain a focus of interest in the treatment of pSS. A host of studies has demonstrated alterations in peripheral and tissue-resident B-cell subsets, genetic and epigenetic modifications in B-cells, and B-cell microRNA expression profiles ^8^. BAFF may promote B-cell over-activation and loss of tolerance in pSS. To date, small studies testing anti-BAFF antibodies in patients with pSS have shown equivocal results ^51– 53^. Two randomized phase II trials of anti-BAFF therapies for pSS are ongoing. In one study, belimumab has been combined with rituximab (ClinicalTrials.gov identifier: NCT02631538; Table 2) on the basis of the rationale that elevated BAFF following B-cell depletion results in selection of self-reactive B-cells during the reconstitution of the B-cell repertoire ^54^. Also, patients in the TEARS study with high baseline BAFF levels had a less robust response to rituximab, raising the possibility that neutralization of BAFF improves the response to rituximab therapy ^55, 56^. In another study, this same pathway was targeted in pSS with ianalumab (VAY736), a monoclonal antibody to the BAFF receptor (BAFF-R) which is expressed on the surface of B-cells. The binding of ianalumab to B-cells blocks BAFF:BAFF-R signaling and also depletes B-cells by direct antibody-dependent cytotoxicity. A small phase II study of a single dose of this agent has demonstrated safety of ianalumab in pSS and provided preliminary evidence of efficacy with a trend toward improvements in the ESSDAI, ESSPRI, and other outcome measures ^57^. A larger phase II trial of ianalumab in pSS is in progress (ClinicalTrials.gov identifier: NCT02962895; Table 2).

Another potential strategy for reducing B-cell activity in pSS is inhibition of PI3Kδ, an intracellular lipid kinase that plays a critical role in B-cell receptor signal transduction ^8^. A selective inhibitor of PI3Kδ, called leniolisib, was recently approved for the treatment of chronic lymphocytic leukemia and non-Hodgkin’s lymphoma. Early studies in pSS have shown upregulation of the PI3Kδ pathway in salivary gland tissue, and murine studies of PI3Kδ inhibition have suggested possible efficacy in reducing glandular invasion by lymphocytes, cytokine production, and autoantibody production ^58^. A double-blind, randomized, placebo-controlled clinical trial of leniolisib showed an acceptable safety profile but no clear signal of efficacy by ESSDAI or patient-reported measures ^59^.

The type I interferon pathway represents a linkage between altered innate and adaptive immunity in pSS. Upregulation of type I interferon-stimulated genes in local tissues and systemic immune cells of patients with pSS results in an increase in BAFF signaling, autoreactive B-cell activity, and autoantibody production ^60^. Several studies have suggested that the strength of the interferon “signature” of patients with pSS might be a criterion for selecting patients most likely to respond to targeted therapies ^61, 62^. So far, direct interferon inhibitors have not yet been tried in pSS. However, Janus kinase (JAK) inhibitors are known to substantially impact the interferon pathway and thus may offer a viable therapeutic strategy in pSS. Early support for this strategy was provided by a study of the JAK inhibitor filgotinib, which has been shown to modify messenger RNA expression of interferon-related genes and the BAFF gene in human salivary gland epithelial cells and salivary gland organoid cultures ^63^. A phase II trial of filgotinib in pSS is ongoing (ClinicalTrials.gov identifier: NCT03100942; Table 2) ^54^.

Although abatacept therapy failed to show clinical efficacy in a recent phase III clinical trial involving patients with pSS, other avenues for inhibiting B- and T-cell co-stimulation remain viable future treatment strategies. Antigen-presenting cells like B-cells and dendritic cells receive activating co-stimulatory signals when their CD40 receptors bind to CD40 ligand on T helper cells. Intriguingly, ductal epithelial cells in patients with pSS also express CD40 ^64^. A recent study by Wieczorek *et al*. demonstrated activation of the CD40 pathway in the salivary gland tissue of a murine model of pSS; inhibiting this pathway led to a reduction in sialadenitis, ectopic lymphoid structure formation, and autoantibody production ^65^. A small phase II clinical trial showed that anti-CD40 antibody in pSS improves the ESSDAI score and other disease measures ^54^. Additional clinical trials to further evaluate this therapy are in progress (ClinicalTrials.gov identifier: NCT03905525; Table 2). Table 2 lists the ongoing phase II and III clinical trials of targeted therapies for pSS from the ClinicalTrials.gov database.

## Conclusion: looking for an oasis in the desert

What progress in the future is likely to facilitate the development of new therapies for pSS? Although the results of recent studies are encouraging, it seems likely that an optimal therapeutic target has not been identified for improving glandular function or significantly modifying systemic disease across multiple domains. Perhaps, targeting the CD40–CD40L pathway will provide demonstrable clinical efficacy in this setting. Others have suggested that targeting neuroendocrine pathways may be more productive for modifying tear and salivary secretion ^66^. Epithelial cells, which are hypothesized to be key drivers of glandular inflammation, demand more attention as therapeutic targets given their capacity to interact with T- and B-cells and secrete pro-inflammatory cytokines. Fine-tuning the study population to achieve a higher likelihood of treatment response represents another strategy for optimizing the detection of treatment responses. Identification of patient subsets on the basis of predictive biomarkers (for example, biomarkers associated with a higher likelihood of treatment response) or mechanism of action of the test drug (for example, subsets with strong interferon signature for an intervention targeting interferon pathways) would be the next step in evolving toward a more personalized treatment approach.

Further refinements in the ESSDAI and other outcome measures may be needed to improve the ability to detect clinically meaningful changes in systemic disease activity. Should pulmonary function studies be routinely included in clinical trials to assess changes in lung function? Can better assessments of arthritis be developed to detect important changes in the activity of joint inflammation? In patients with pSS, unlike in patients with RA, swollen joints are the exception to the rule; joint counts may prove less sensitive to change compared with their responsiveness in patients with active RA. The field could develop more sensitive patient-reported outcome measures, which, for example, might leverage internet-based mobile devices for ascertainment of time-integrated data related to the burden of fatigue and joint pain. The prospects for the discovery of the first disease-modifying therapy for pSS are indeed bright with the entry of an increasing number of exciting new agents into the pipeline.

## Abbreviations

ACR, American College of Rheumatology; AECG, American-European Consensus Group; BAFF, B-cell activating factor; BAFF-R, B-cell activating factor receptor; CA6, carbonic anhydrase 6; ESSDAI, EULAR Sjögren’s syndrome Disease Activity Index; ESSPRI, EULAR Sjögren’s Syndrome Patient-Reported Index; EULAR, European League Against Rheumatism; JAK, Janus kinase; JOQUER, Randomized Evaluation of hydroxychloroquine in primary Sjögren’s syndrome; LT, lymphotoxin; PSP, parotid secretory protein; pSS, primary Sjögren’s syndrome; RA, rheumatoid arthritis; SICCAC, Sjögren’s International Collaborative Clinical Alliance Cohort; SLE, systemic lupus erythematosus; SP1, salivary protein 1; TEARS, Tolerance and Efficacy of Rituximab in primary Sjögren’s syndrome; TRACTISS, Trial of Anti-B cell Therapy in Patients with primary Sjögren’s syndrome; VAS, visual analogue scale
